# Structural changes in the retina as a potential biomarker in Parkinson's disease: an approach from optical coherence tomography

**DOI:** 10.3389/fnimg.2024.1340754

**Published:** 2024-03-01

**Authors:** Santiago Poveda, Ximena Arellano, Oscar Bernal-Pacheco, Alejandro Valencia López

**Affiliations:** ^1^Department of Neurology, Central Military Hospital, Bogotá, Colombia; ^2^Department of Ophthalmology, Central Military Hospital, Bogotá, Colombia; ^3^Roosevelt Orthopedic Institute, Bogotá, Colombia; ^4^Fundación Santa Fe de Bogotá University Hospital, Bogotá, Colombia; ^5^University of Manizales, Caldas, Colombia

**Keywords:** Parkinson's disease, neurodegenerative diseases, optical coherence tomography, thickness of the nerve fiber layer, ganglion cell complex

## Abstract

**Introduction:**

Parkinson's disease (PD) presents challenges in early diagnosis and follow-up due to the lack of characteristic findings. Recent studies suggest retinal changes in PD are possibly indicative of neurodegeneration. We explored these changes using optical coherence tomography (OCT) to assess retinal nerve fiber layer (RNFL) and ganglion cell complex (GCC) thickness.

**Methods:**

Thirty PD and non-PD patients were matched according to demographic characteristics and OCT and clinical evaluations to rule out other neurodegenerative and visual diseases.

**Results:**

We observed a significant thinning of the RNFL in patients diagnosed with PD compared to non-PD patients (*p* = 0.015). Additionally, this reduction in RNFL thickness was found to correlate with the severity of the disease (*p* = 0.04).

**Conclusion:**

The OCT serves as a tool for quantifying neurodegeneration in PD, showing a significant correlation with disease severity. These findings suggest that OCT could play a crucial role as a potential biomarker in the diagnosis and monitoring of PD.

## Introduction

Neurodegenerative diseases are increasing as the population ages, with idiopathic Parkinson's disease (PD) emerging as the second most common after Alzheimer's disease and displaying the fastest global growth (Saavedra Moreno et al., 2019). Dopaminergic denervation constitutes a key aspect of the pathophysiology, elucidating the primary motor symptoms of the disease, namely, bradykinesia, rest tremor, and rigidity. Nevertheless, the extensive range of the differential diagnosis can lead to delays, particularly in the absence of distinctive findings in early-stage complementary tests before the manifestation of motor symptoms (Satue et al., 2014). Moreover, Parkinson's disease (PD) encompasses prodromal non-motor symptoms, encompassing autonomic, neuropsychiatric, and sleep-related manifestations. Recognizing these symptoms is crucial for a comprehensive understanding of the disease's pathophysiology and facilitating early diagnosis (Sari et al., 2015).

Dopamine is also identified within the retina, where it is released by a distinctive group of amacrine cells situated in the proximal inner nuclear layer. These cells extend projections to both the inner and outer plexiform layers (Satue et al., 2014). Dopamine, facilitated by the permeability of gap junctions, promotes the interaction between rod and cone photoreceptors with horizontal cells, and consequently, with bipolar cells. This process is inhibited by the dopamine D2 receptor family. On the other hand, ganglion cells, bipolar cells, horizontal cells, and amacrine cells are stimulated by dopamine D1 receptors (Mohana Devi et al., 2020).

Postmortem and *in vivo* studies have substantiated the common occurrence of retinal accumulation of alpha-synuclein in individuals with Parkinson's disease (PD; Guo et al., 2018). The depletion of dopamine at the level of ganglion cells and amacrine cells elucidates how this process can lead to aberrant inputs to the visual cortex. This, in turn, gives rise to alterations in functions such as visuospatial construction, object perception, face recognition, and emotional processing. Additionally, it impacts visual acuity, contrast sensitivity, color vision, and motion perception, contributing to phenomena such as visual hallucinations (Inzelberg et al., 2004; Garcia-Martin et al., 2012; Nicolas Alves et al., 2023).

The observations regarding retinal structures in Parkinson's disease (PD) have been substantiated through optical coherence tomography (OCT), revealing a reduction in the thickness of the nerve fiber layer, primarily involving non-myelinated axons of retinal ganglion cells (Obis et al., 2018; Mailankody et al., 2019); non-invasive techniques, which allow obtaining *in vivo* images of the retina, measure its layers, providing evidence of alterations that may be related to loss of retinal dopamine (Lee et al., 2022). Optical coherence tomography (OCT) of the optic nerve serves as a marker for inflammation and neurodegeneration in various diseases, including multiple sclerosis, neuromyelitis optica, amyotrophic lateral sclerosis, and Huntington's disease (Vujosevic et al., 2023). Due to the concurrent effects of normal aging observed in patients with Parkinson's disease (PD), the presence of mature cataracts, glaucoma, and conditions affecting retinal or macular vessels may influence the reliability of examinations. Consequently, this can impact the specificity of predictive values associated with anatomical changes in the retina (Nicolas Alves et al., 2023).

The hypothesis of the study is that OCT measurements, particularly the thinning of the RNFL and GCC thickness, could be valuable indicators of neurodegeneration associated with Parkinson's disease. The research aims to establish connections between these retinal changes and relevant clinical variables, such as disease severity and levodopa treatment, with the goal of advancing the understanding and early diagnosis of PD.

## Methodology

We conducted an observational, retrospective study approved by the Ethics Committee of the Central Military Hospital, Colombia. The study focused on evaluating OCT findings of the macula and optic nerve in Parkinson's disease (PD) patients at the Central Military Hospital in Bogotá, Colombia, between 2021 and 2022. We employed a non-probabilistic convenience sampling strategy. The inclusion criteria for this study encompassed:

- Patients diagnosed with Parkinson's disease aged between 45 and 85 years.
- Absence of any other neurodegenerative disease.
- Montreal Cognitive Assessment (MoCA) score > 23.
- Intraocular pressure < 21 mmHg.
- No conditions affecting the retina and optic nerve such as glaucoma, macular degeneration, diabetic retinopathy, or HIV-related retinopathy.
- No history of ocular trauma or visual field alterations.
- Absence of posterior pole pathology like macular degeneration and no opacity of ocular media such as cataracts.
- No prior ophthalmic surgery except uncomplicated cataract surgery.
- Refractive errors < 5 diopters spherical or < 3 diopters cylindrical.

Non-compliance with the inclusion criteria constituted the exclusion criteria. For establishing reference OCT parameters, individuals without Parkinson's disease (non-PD), matched for age and sex, with the exclusion factors ruled out, were selected as the non-PD patient group.

The sociodemographic profiling and characterization of Parkinson's disease (PD) patients encompassed details such as disease duration, treatment specifics, and the equivalent dose of levodopa. Patients were clinically assessed by a movement disorders specialist, through structured anamnesis and a comprehensive neurological examination, including the Unified Parkinson's Disease Rating Scale Part III (UPDRS III) and the Hoehn and Yahr classification (Goetz et al., 2008). Subsequently, the OCT findings were outlined for both PD and non-PD patients, followed by an evaluation of the relationship between clinical variables and OCT results. It is important to note that OCT measurements from both eyes of each patient were averaged to obtain a single data point for analysis.

The analysis was structured into three specific axes. In the first axis, focused on PD patients, an initial check for normal distribution using the Shapiro–Wilk test was performed on quantitative variables. Pearson correlation was employed for variables conforming to a normal distribution, with Spearman correlation utilized for those deviating from normality. The correlation analysis encompassed variables such as disease duration, UPDRS III scores, and equivalent levodopa dose. Correlation values of at least +/- 0.2 were considered low, and those above +/- 0.4 were deemed moderate. Given the correlation of several clinical variables with those obtained from OCT measurements, we implemented the statistical Bonferroni adjustment method to correct for multiple comparisons. This rigorous statistical approach ensures the reliability and accuracy of our findings by accounting for the potential inflation of type I error rates that can arise when conducting multiple tests simultaneously. Further comparisons involved assessing each optical coherence tomography (OCT) variable with Hoehn and Yahr classification, utilizing *t*-tests for parametric PD data and *U* Mann-Whitney for non-parametric PD data. Descriptive analysis encompassed estimating various statistics for both quantitative and categorical PD variables. The second axis involved comparing PD cases with non-PD controls, assessing normality using the Shapiro–Wilk test and conducting paired analysis, nested by sex and age. Paired *t*-tests and Wilcoxon signed-rank tests were applied for normally and non-normally distributed PD variables, respectively. Frequency comparison was conducted for categorical PD variables. All analyses were carried out at a 95% confidence level using Stata v14 (StataCorp) and IBM SPSS Statistics V22. A significance level of *P* < 0.05 was considered statistically significant.

## Results

Data from 30 Parkinson's disease (PD) patients, comprising 60% women and 40% men with a mean age of 62.6 years +/- 7.5 SD, were analyzed. PD severity, assessed by the Hoehn and Yahr scale, ranged from stages 1 to 3, with a median of 2. The average disease duration was 7.5 years +/- 5.7 SD. Motor symptoms evaluated using the UPDRS III scale had an average score of 33.1 points +/- 14.5 SD, with 73.3% of patients in the ON state. The levodopa equivalent dose averaged 741.1 mg +/- 320.1 SD per patient, and 50% of participants underwent deep brain stimulation (DBS) treatment (Table 1).

**Table 1:** Clinical characteristics of patients with Parkinson's disease.

***n*** = **30 patients**		**Media (SD)**	**Range**
Age (y)		62.7 (7.5)	46–80
Duration of illness (y)		7.5 (5.7)	1–30
Levodopa equivalent dose (mg)		741.1 (320.1)	0–1, 450
UPDRS III		33.1 (14.5)	8–67
		***N***	**%**
Sex	Women	18	60
**Hoehn and Yahr classification**			
1		1	3.3
2		16	53.3
3		12	40
4		0	0
5		1	3.33
**Treatment**			
Levodopa		23	76.7
Dopamine agonists	Rotigotine	12	40
	Pramipexole	13	43.3
MAO-B inhibitors	Rasagiline	8	26.7
	Safinamide	3	10
COMT inhibitors	Entacapone	4	13.3
Amantadine		29	96.7
DBS		15	50

y, years; SD, standard deviation; UPDRS III, Unified Parkinson's Disease Rating Scale Part III; MAO-B, monoamine oxidase B; COMT, catechol-o-methyl-transferase; DBS, deep brain stimulation.

For the analysis involving the PD and non-PD patient groups, age and sex were successfully matched in a 1:1 ratio, resulting in 30 patients in each group. In each cohort, 12 patients (40%) were male and 18 patients (60%) were female, with an average age of 62.7 years in the PD group and 62.2 years in the non-PD group, confirming effective pairing. When assessing the characteristics of the macula and optic nerve using OCT in PD patients compared to the non-PD group, a significant decrease was observed in the RNFL with *p* = 0.015 (Table 2).

**Table 2:** Characteristics of optical coherence tomography of the macula and optic nerve.

	**PD (*****n*** = **30)**		**Non-PD (*****n*** = **30)**		***p*-value**
	**Median**	**IQR**	**Median**	**IQR**	
Central foveal thickness (μm)	247.5	(235.5–264)	254	(238–275.5)	0.084
RNFL thickness (μm)	93.5	(81.0–100)	96	(91–102)	0.015
CCG thickness (μm)	80	(74–83.5)	82.5	(78–85.5)	0.064

IQR, interquartile range; RNFL, retinal nerve fiber layer; GCC, ganglion cell complex.

In the initial correlation analyses between clinical variables of PD and OCT parameters, a statistically significant difference was found in the levodopa equivalent dose and the UPDRS III scale. However, upon applying multiple variable correction, only the inverse correlation in RNFL thickness with the UPDRS III scale remained significant, with *p* = 0.04 (Table 3). In the comparison of OCT findings with the Hoehn and Yahr stages of disease as well as disease duration, no significant differences were observed.

**Table 3:** Correlation in the group of patients with Parkinson's disease of OCT parameters with neurological characterization variables.

***n*** = **30**		**Duration of disease**	**LED**	**UPDRS III**
Central thickness	Correlation coefficient	−0.13	−0.29	−0.0001
	Significance	0.5	0.13	0.99
	Bonferroni adjustment	1	0.78	1
RNFL thickness	Correlation coefficient	−0.1	−0.37	−0.49
	Significance	0.59	0.04	0.007
	Bonferroni adjustment	1	0.29	0.04^*^
GCC thickness	Correlation coefficient	−0.10	−0.24	−0.41
	Significance	0.58	0.21	0.02
	Bonferroni adjustment	1	1	0.15

LED, levodopa equivalent dose; RNFL, retinal nerve fiber layer; GCC, ganglion cell complex.

^*^p < 0.05. OCT values correspond to the average of the right and left eye.

## Discussion

PD patients exhibit thinner retinas compared to age and gender-matched non-PD patients; this study revealed a significant decrease in the thickness of the retinal nerve fiber layer (RNFL). While some authors have reported the absence of changes in OCT studies (Chorostecki et al., 2015; Aydin et al., 2018), the most recent trend robustly indicates an increase in thinning, particularly affecting the inner layers of the retina, including the retinal nerve fiber layer (RNFL) and ganglion cell complex (GCC; Inzelberg et al., 2004; Garcia-Martin et al., 2012; Yu et al., 2014; Sari et al., 2015; Aydin et al., 2018; Sengupta et al., 2018; Unlu et al., 2018; Chrysou et al., 2019; Zou et al., 2020; Huang et al., 2021; Zhou et al., 2021; Kamata et al., 2022; Kundu et al., 2023).

From a pathophysiological perspective, it is anticipated that with an extended duration of the disease, progressive dopaminergic depletion would account for a directly proportional reduction in retinal thickness. This could potentially serve as a biological marker of neurodegeneration through OCT. Several studies substantiate this theory (El-Kattan et al., 2022; Wang et al., 2022), especially when discriminating the analysis by quadrants and with a disease duration exceeding 10 years (Lee et al., 2014a; Satue et al., 2017); however, in the current study, the duration of the disease had no impact on the modification of retinal RNFL or GCC thickness. Although this lack of correlation has also been previously described (Inzelberg et al., 2004; Roth et al., 2014; Sari et al., 2015; Aydin et al., 2018), it has not been expressed in terms of its specific location by quadrants. The average duration of the disease was less than a decade (8 years), which could potentially influence this lack of correlation.

The levodopa equivalent dose (LED) indirectly reflects the severity of motor impairment in PD. In this study, the equivalent dose of levodopa demonstrated a weak inverse correlation with the thickness of the retinal nerve fiber layer (RNFL) and ganglion cells (GCC), as noted in other publications (Lee et al., 2014b; Zhang et al., 2021). Despite this weak correlation, it becomes non-significant after applying multiple variable correction.

The neurological examination, administered using UPDRS III under medication while patients were in an ON state, was conducted in 73% of the population, posing potential challenges in interpreting the findings. In our study, an inverse correlation was observed in the thickness of the retinal nerve fiber layer (RNFL) and the retinal ganglion cell complex (CCG), similar to findings in two studies that encompassed a comparable population group and a meta-analysis (Satue et al., 2014). Although the medication ON or OFF status was not reported in most studies, this omission could potentially account for why some studies found no correlation with the thickness of the retinal nerve fiber layer (RNFL) and ganglion cell complex (GCC) layers (Aydin et al., 2018; Elanwar et al., 2023). Upon the implementation of multiple comparisons correction, a statistically significant correlation is observed, revealing a marked decrease in RNFL thickness as UPDRS III scores increase.

The findings from our study regarding disease severity suggest that, with higher levodopa dose requirements and increased motor compromise, discernible changes in retinal structure manifest through pronounced thinning of the retina. This phenomenon holds potential as a biomarker for the disease.

## Conclusion

In patients with Parkinson's disease, degenerative processes extend beyond the brain, manifesting as retinal neurodegeneration characterized by increased axonal damage, which correlates with greater disease severity as measured by UPDRS III and a higher levodopa equivalent dose. From this perspective, assessing axonal damage through the measurement of retinal nerve fiber layer (RNFL) and ganglion cell complex (GCC) thickness via optic nerve OCT can play a role as a biomarker in Parkinson's disease.

## Data availability statement

The original contributions presented in the study are included in the article/supplementary material, further inquiries can be directed to the corresponding authors.

## Ethics statement

The studies involving humans were approved by Ethics Committee Central Military Hospital, Bogotá, Colombia. The studies were conducted in accordance with the local legislation and institutional requirements. The participants provided their written informed consent to participate in this study. Written informed consent was obtained from the individual(s) for the publication of any potentially identifiable images or data included in this article.

## Author contributions

SP: Writing – original draft. XA: Writing – original draft. OB-P: Writing – review & editing. AV: Writing – original draft.
